# Systemic and adipocyte transcriptional and metabolic dysregulation in idiopathic intracranial hypertension

**DOI:** 10.1172/jci.insight.145346

**Published:** 2021-05-24

**Authors:** Connar S.J. Westgate, Hannah F. Botfield, Zerin Alimajstorovic, Andreas Yiangou, Mark Walsh, Gabrielle Smith, Rishi Singhal, James L. Mitchell, Olivia Grech, Keira A. Markey, Daniel Hebenstreit, Daniel A. Tennant, Jeremy W. Tomlinson, Susan P. Mollan, Christian Ludwig, Ildem Akerman, Gareth G. Lavery, Alexandra J. Sinclair

**Affiliations:** 1 Institute of Metabolism and Systems Research, College of Medical and Dental Sciences, University of Birmingham, Birmingham, United Kingdom.; 2 Centre for Endocrinology, Diabetes and Metabolism, Birmingham Health Partners, Birmingham, United Kingdom.; 3 Institute of Inflammation and Ageing, College of Medical and Dental Sciences, University of Birmingham, Birmingham, United Kingdom.; 4 Department of Neurology, University Hospitals Birmingham National Health Service (NHS) Foundation Trust, Queen Elizabeth Hospital, Birmingham, United Kingdom.; 5 School of Life Sciences, University of Warwick, Coventry, United Kingdom.; 6 Upper GI Unit and Minimally Invasive Unit, Heartlands Hospital, University Hospitals Birmingham NHS Foundation Trust, Birmingham United Kingdom.; 7 Oxford Centre for Diabetes, Endocrinology & Metabolism, National Institute for Health Research (NIHR) Oxford Biomedical Research Centre, University of Oxford, Churchill Hospital, Headington, Oxford, United Kingdom.; 8 Birmingham Neuro-Ophthalmology, Ophthalmology Department, Queen Elizabeth Hospital, University Hospitals Birmingham NHS Foundation Trust, Birmingham, United Kingdom.

**Keywords:** Neuroscience, Ophthalmology, Neurological disorders, Obesity

## Abstract

**BACKGROUND:**

Idiopathic intracranial hypertension (IIH) is a condition predominantly affecting obese women of reproductive age. Recent evidence suggests that IIH is a disease of metabolic dysregulation, androgen excess, and an increased risk of cardiovascular morbidity. Here we evaluate systemic and adipose specific metabolic determinants of the IIH phenotype.

**METHODS:**

In fasted, matched IIH (*n* = 97) and control (*n* = 43) patients, we assessed glucose and insulin homeostasis and leptin levels. Body composition was assessed along with an interrogation of adipose tissue function via nuclear magnetic resonance metabolomics and RNA sequencing in paired omental and subcutaneous biopsies in a case-control study.

**RESULTS:**

We demonstrate an insulin- and leptin-resistant phenotype in IIH in excess of that driven by obesity. Adiposity in IIH is preferentially centripetal and is associated with increased disease activity and insulin resistance. IIH adipocytes appear transcriptionally and metabolically primed toward depot-specific lipogenesis.

**CONCLUSION:**

These data show that IIH is a metabolic disorder in which adipose tissue dysfunction is a feature of the disease. Managing IIH as a metabolic disease could reduce disease morbidity and improve cardiovascular outcomes.

**FUNDING:**

This study was supported by the UK NIHR (NIHR-CS-011-028), the UK Medical Research Council (MR/K015184/1), Diabetes UK, Wellcome Trust (104612/Z/14/Z), the Sir Jules Thorn Award, and the Midlands Neuroscience Teaching and Research Fund.

## Introduction

Idiopathic intracranial hypertension (IIH) is characterized by elevated intracranial pressure (ICP) and papilledema typically manifesting as disabling daily headaches and visual disturbances, which leads to permanent visual loss in up to 25% of patients (1–4). IIH predominantly occurs in obese women of reproductive age (greater than 90%), where incidence is increasing in line with the obesity epidemic (5). Consequently, IIH constitutes a substantial financial burden in the clinical setting (5). IIH incidence increases with rising body mass index (BMI), with disease activation often occurring following rapid weight gain (6, 7). Weight loss is therapeutic in IIH, with reduction in adiposity associated with reduction in ICP and improvements in headache and visual outcomes, suggesting a role of adipose tissue in IIH pathogenesis (8, 9).

The etiology of IIH remains unclear and an unmet research priority (10). IIH is no longer regarded as a disease isolated to the CNS because patients with IIH have double the risk of cardiovascular disease (CVD) compared with obese individuals (6, 11). Importantly, IIH is a condition of androgen excess, and like polycystic ovarian syndrome, may contribute to cardiometabolic diseases, including type 2 diabetes mellitus (T2D) and CVD (12–14). We hypothesize that metabolic perturbations, possibly emanating from adipose tissue, contribute to the increased CVD risk in IIH.

In a large cohort of female patients with active IIH, we assessed markers of systemic metabolic dysregulation and analyzed adipose tissue distribution. We also aimed to identify underpinning molecular mechanisms through investigation of a unique cohort of patients with IIH from which we investigated paired omental (OM) and subcutaneous (SC) adipose tissue function. Here we define what we believe to be the first detailed metabolic phenotype of IIH and identify molecular mechanisms within adipose tissue that may contribute to CVD risk. We demonstrate that patients with IIH have dysregulated systemic metabolism, in excess of that mediated by obesity, being more insulin resistant in the context of hyperleptinemia and adipocyte leptin hypersecretion. Furthermore, SC adipose would appear to be transcriptionally primed for increased calorie intake with a unique depot-specific lipogenic profile.

## Results

### Patient characteristics.

Patients with active untreated IIH (*n* = 97) and control patients (*n* = 43) were prospectively recruited. The IIH cohort were all female, aged 32.4 ± 7.8 years, and obese (BMI 40.0 ± 6.5 kg/m^2^), with raised lumbar puncture opening pressure (LP OP) (34.8 ± 5.7 cm cerebrospinal fluid, cmCSF) and papilledema. The control cohort were matched for BMI and sex but were older (Table 1). Baseline characteristics and characteristics of the substudy cohorts can be found in Table 1 and Supplemental Figure 1; supplemental material available online with this article; https://doi.org/10.1172/jci.insight.145346DS1

### IIH patients have an insulin-resistant phenotype.

**Figure 1:**
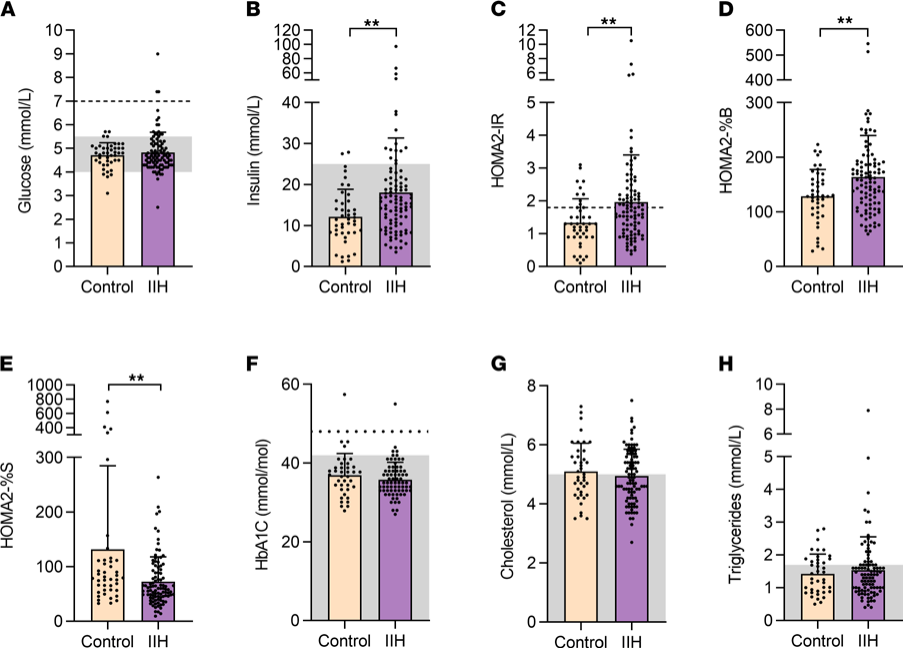
Perturbed metabolic function in IIH. Histograms of fasted glucose (**A**), insulin (**B**), HOMA2-IR (**C**), HOMA2-%B (**D**), HOMA2-%S (**E**), HbA1c (**F**), cholesterol (**G**), and triglycerides (**H**) in control (*n* = 43) and IIH patients (*n* = 97). Gray boxes represent healthy clinical reference ranges. (**A** and **F**) Dotted lines represent thresholds suggestive of type 2 diabetes mellitus. (**C**) Dotted line represents HOMA2-IR score 1.8, threshold for insulin resistance. *n* represents an individual patient. Data presented as mean ± SD, **P < 0.01.

Epidemiological data have suggested that patients with IIH have an increased risk of T2D and cardiovascular morbidity, suggestive of systemic metabolic dysfunction (6). Hence, we evaluated markers of insulin resistance in IIH. Fasting insulin levels were elevated in IIH (IIH 18.1 ± 13.3 mIU/L vs. controls 12.1 ± 6.7 mIU/L, Mann-Whitney *U* test, *U* = 1421, *P* = 0.0025, Figure 1B), with markers of insulin resistance elevated in IIH compared with controls (HOMA2-IR, 1.97 ± 1.44 vs. 1.33 ± 0.74; *P* = 0.0030, Figure 1C), higher β cell function in IIH (HOMA2-%B scores of 163.6 ± 76.4 vs. 128.6 ± 48.7; *P* = 0.0097, Figure 1D), and lower insulin sensitivity in IIH (HOMA2-%S scores of 72.4 ± 45.5 vs. 131.6 ± 153.3; *P* = 0.0030, Figure 1E), and these features are associated with a progression to T2D (15). Additionally, a higher proportion of patients with IIH had insulin resistance, as defined by a HOMA2-IR more than 1.8, (IIH 47.5% vs. controls 22.6%; *P* = 0.0155, Fisher’s exact test) (16). Given that the control cohort was older than the IIH cohort, a multiple regression analysis was carried out on the HOMA2-IR results, taking both age and BMI into consideration. This analysis demonstrated that patients with IIH had higher levels of insulin resistance (1.89 ± 0.28 vs. 1.51 ± 0.26 HOMA2-IR; *P* < 0.0001, Supplemental Figure 2). The fasting glucose (Figure 1A) and HbA1c (Figure 1F) levels were comparable between IIH and control subjects, despite the controls being older. The relationship between insulin resistance and IIH disease activity, inferred from the ICP, was evaluated, where the relationship between LP OP and HOMA2-IR was weak (*P* = 0.051, correlation coefficient *ρ* = 0.12).

We evaluated the lipid profile in IIH, a risk factor for cardiovascular morbidity (17). We demonstrated no differences in IIH fasted cholesterol (IIH 4.94 ± 0.91 vs. controls 5.09 ± 0.95 mmol/L, *t* test, *t*_133_ = 0.88, *P* = 0.37, Figure 1G) and triglycerides (IIH 1.54 ± 1.01 vs. controls 1.42 ± 0.60 mmol/L, Mann-Whitney *U*, *U* = 1889, *P* = 0.96, Figure 1H) compared to the controls. We also evaluated hepatic and renal function in the IIH and control subjects (Substudy 1, Table 1) and noted a number of differences in IIH, although levels remained within the normal clinical reference ranges.

### Obesity in IIH.

**Figure 2:**
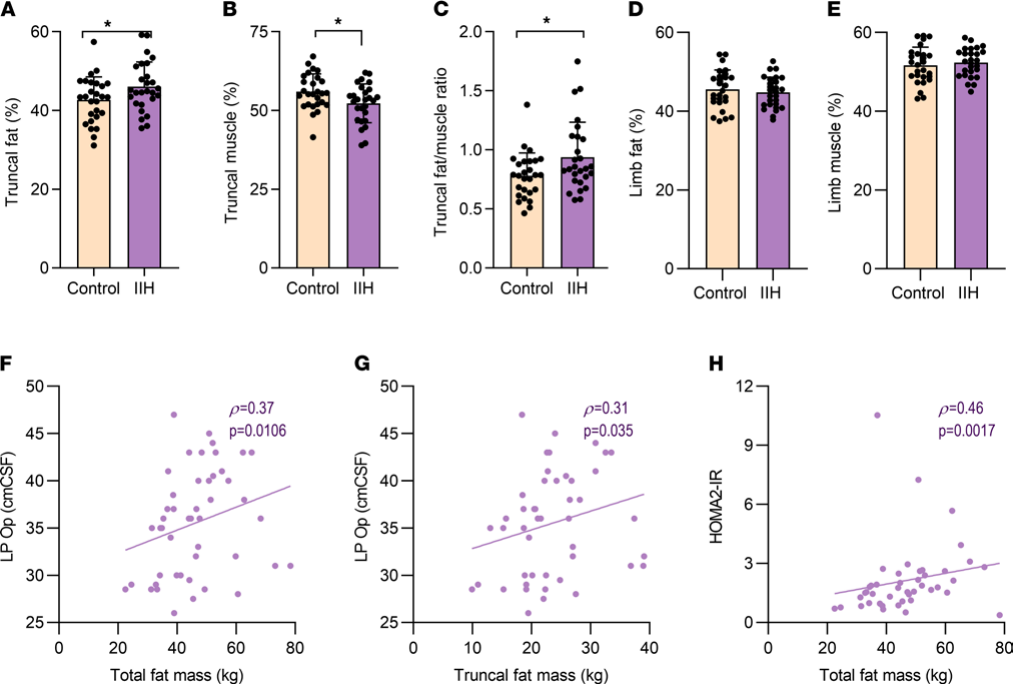
IIH patients have an altered body composition. IIH and control patient body composition assessed via dual-energy x-ray absorptiometry (DEXA) scanning. Histograms of (**A**) truncal fat percentage, (**B**) truncal lean percentage, (**C**) truncal fat/lean ratio, (**D**) limb fat percentage, and (**E**) limb lean percentage. (**A**–**E**) *n* = 27 for control and IIH. Scatter graphs of LP OP versus (**F**) total body fat (*n* = 47) and (**G**) truncal fat mass (*n* = 47) and (**H**) HOMA2-IR versus total fat mass (*n* = 44) in IIH patients. *n* represents an individual patient. Unpaired *t* test (**B**–**D**). Mann-Whitney *U* test (**A** and **E**). Spearman’s correlations (**F**–**H**). Data presented as mean ± SD. *P < 0.05.

It is well established that adipose distribution is a determinant of insulin resistance and cardiovascular risk (18, 19). Given that our data demonstrated a greater degree of insulin resistance, we assessed the adipose distribution and lean mass in patients with IIH (Substudy 2, Table 1) (20). We determined that patients with IIH had a similar total mass, fat mass, and lean mass compared to control patients. However, when assessing the truncal region, the adipose depot most associated with metabolic risk and insulin resistance, we demonstrated increased fat (IIH 46.03 ± 6.29 vs. controls 42.66 ± 5.84 %, *t* test, *t*_52_ = 2.037, *P* = 0.046, Figure 2A) and lower truncal lean mass (IIH 52.27 ± 6.15 vs. controls 55.97 ± 5.64%, *t* test, *t*_52_ = 2.304, *P* = 0.025, Figure 2B) in IIH. Accordingly, there was an increased truncal fat to lean mass ratio in IIH patients (0.94 ± 0.30 vs. 0.78 ± 0.19, Mann-Whitney *U*, *U* = 24, *P* = 0.0441; Figure 2C). That there were no differences in limb fat (Figure 2D) and lean mass (Figure 2E) suggests preferential truncal adipose deposition in IIH.

### Obesity is associated with insulin resistance and ICP.

Previous work demonstrated that truncal adiposity correlates with LP OP in IIH, suggesting that adiposity is associated with IIH disease activity (8). In this larger IIH cohort we recapitulated this finding, demonstrating that LP OP correlated with total (*P* = 0.016, *P* = 0.37, Figure 2F) and truncal fat mass (*P* = 0.035, *P* = 0.31, Figure 2G). Total adipose mass is strongly associated with insulin resistance in obese individuals and was observed in the present IIH cohort, linking excess abdominal adiposity to insulin resistance in the IIH patients (*P* = 0.0017, *P* = 0.46, Figure 2H) (20).

### IIH patients have comparative hyperleptinemia.

The satiety adipokine leptin is strongly associated with obesity, insulin resistance, and metabolic dysfunction and has been proposed to be pathogenic in IIH (21, 22). Interpretation of previous leptin studies in IIH is limited by sample size, selection of the control cohorts, and variable fasting status (21, 23–26). Here we compared both serum and CSF in patients with IIH against a healthy control cohort to determine if leptin was altered and assessed the relationship of leptin to markers of metabolic dysfunction.

**Figure 3:**
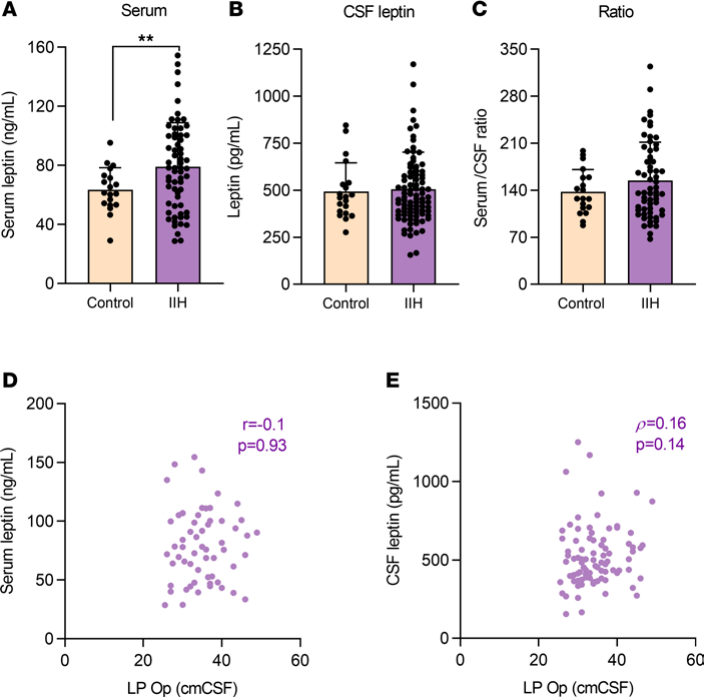
IIH patients display an enhanced hyperleptinemia. Fasted leptin levels assessed by ELISA in IIH and control patients. Serum leptin (**A**) in control (*n* = 19) and IIH patients (*n* = 60). CSF leptin in IIH (*n* = 87) and control (*n* = 20) patients (**B**). Serum/CSF ratio in control (*n* = 19) and IIH (*n* = 58) patients (**C**). Scatter graph of LP OP versus serum leptin (**D**) and CSF leptin (**E**). *n* represents an individual patient. Welch’s *t* test (**A**) and Mann-Whitney *U* test (**B** and **C**). Pearson’s correlation (**D**) and Spearman’s correlation (**E**). Data presented as mean ± SD, **P < 0.01.

In the study cohort, we demonstrated elevated fasted serum leptin (IIH 79.5 ± 30.4 vs. controls 63.5 ± 14.9 ng/mL, Welch’s *t* test *t*_63.09_ = 3.059; *P* = 0.003, Figure 3A) in patients with IIH compared with patients with obesity (matched sex and BMI) (Supplemental Figure 1). A further sensitivity analysis, additionally matching the controls for age as well as sex and BMI, confirmed raised serum leptin (IIH 75.6 ± 30.4 vs. controls 63.5 ± 14.9, Welch’s *t* test, *t*_59.20_ = 2.089, *P* = 0.04, Supplemental Figure 3A). This demonstrates that patients with IIH had hyperleptinemia in excess of that observed in obesity (27). Transport of leptin into the CNS is saturable at higher levels of serum leptin in obesity (28). In keeping with this there were no differences in CSF and serum/CSF ratio between IIH and control subjects (Figure 3, B and C; and Supplemental Figure 3, B and C). It is unlikely that hyperleptinemia is driving disordered CSF dynamics in IIH because neither serum (Figure 3D) nor CSF (Figure 3E) leptin levels correlated with LP OP in IIH.

IIH hyperleptinemia is likely a reflection of systemic metabolic dysregulation in IIH. We note that body fat percentage positively correlates with serum leptin in IIH patients (*P* < 0.0001, *r* = 0.74) as it does in obesity (28). Additionally, elevated serum leptin is known to impact β cell function in obesity, and in keeping with this, serum leptin is associated with β cell function in IIH (HOMA-%B, *P* = 0.029, *r* = 0.33) (29).

### Assessment of paired SC and OM adipose tissue.

We showed that adiposity is associated with LP OP, a reflection of IIH disease activity (Figure 2). Loss of adiposity is disease modifying in IIH (8, 9). We have also demonstrated hyperleptinemia in IIH in excess of that driven by obesity (Figure 3). In obesity, these factors are associated with disordered adipose tissue metabolism (27). Consequently, we hypothesized that in IIH, adipose tissue would display perturbed function and metabolism, in excess of that expected from obesity alone. In order to examine our hypothesis in detail, we recruited IIH and control subjects undergoing bariatric surgery and collected matched abdominal SC and OM adipose tissue biopsies (patients matched for age, sex, and BMI; Supplemental Figure 4) and conducted a series of in vitro, molecular, and metabolic assessments to examine phenotypes.

### SC and OM tissue morphology.

**Figure 4:**
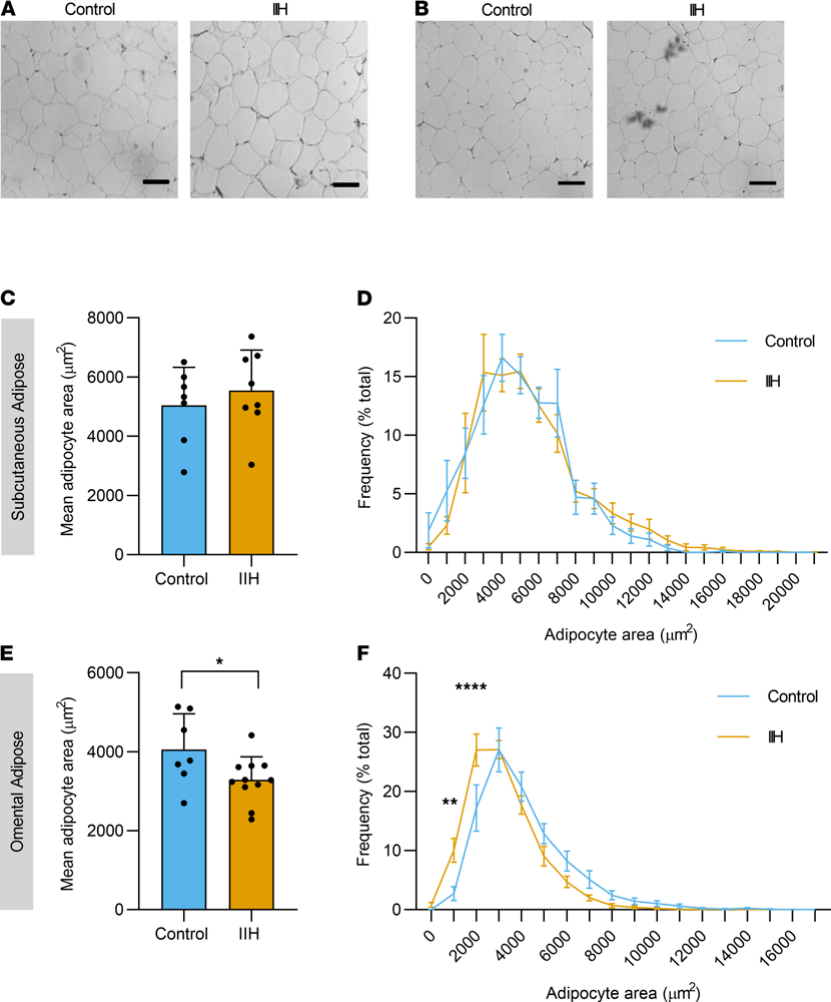
Histomorphometric analysis of IIH adipose tissue. Micrographs of paired subcutaneous (SC) (**A**) and omental (OM) (**B**) adipose tissue from age-, sex-, and BMI-matched control and IIH patients. Mean SC adipocyte area (**C**) and adipocyte area frequency (**D**) adipocyte area in control (*n* = 7) and IIH (*n* = 8). Mean OM adipocyte area (**E**) and adipocyte area frequency (**F**) in control (*n* = 7) and IIH (*n* = 11). *n* represents an individual patient. Scale bar: 100 μm. Unpaired *t* test (**C** and **E**). Two-way ANOVA with Sidak’s multiple-comparison test (**D** and **F**). Data presented as mean ± SD (**C** and **E**) and mean ± SEM (**D** and **F**), *P < 0.05, **P < 0.01, ****P < 0.0001.

Histomorphometric analysis compared adipose tissue (SC and OM adipose depots) between IIH and control subjects (matched age, sex, and BMI; Supplemental Figure 4). IIH SC adipocytes (Figure 4C) had a similar cross-sectional area compared to controls, with a similar distribution of adipocyte area (Figure 4D). However, IIH OM adipocytes were smaller than controls (IIH 3286 ± 176 vs. controls 4056 ± 342 μm^2^; *t* test *t*_16_ = 2.206, *P* = 0.042, Figure 4E). In particular, there was an increased frequency of adipocytes at an area of 1000 μm^2^ (IIH 8.8 ± 1.7 vs. controls 2.7 ± 1.2%, *P* < 0.01) and of 2000 μm^2^ (IIH 27.9 ± 2.6 vs. controls 17.2 ± 3.9%; *P* < 0.0001, Figure 4F), indicating an increased proportion of small adipocytes (ordinary 2-way ANOVA followed by Sidak’s test, row factor *F*_17,288_ = 87.82, *P* < 0.0001).

### Adipocyte leptin hypersecretion in IIH.

**Figure 5:**
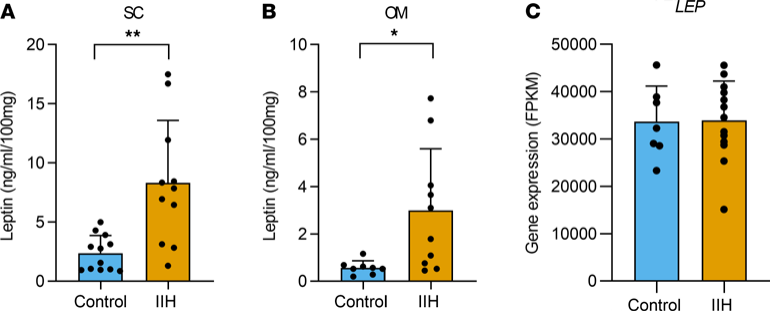
Adipocyte leptin hypersecretion in IIH. Leptin secretion assessed from ex vivo adipose tissue via ELISA in control and IIH patients. (**A**) Leptin secretion from SC adipose tissue in controls (*n* = 12) and IIH (*n* = 11). (**B**) Leptin secretion from OM adipose tissue in controls (*n* = 8) and IIH (*n* = 10). (**C**) *LEP* gene expression in subcutaneous adipose tissue. *n* represents an individual patient. Welch’s *t* test (**A** and **B**), *t* test (**C**). Data presented as mean ± SD, *P < 0.05, **P < 0.01. LEP, leptin; FPKM, fragments per kilobase million.

We showed in Figure 3 that IIH is a state of hyperleptinemia. Consequently, we assessed leptin secretion in ex vivo adipose tissue (IIH compared with matched controls, Supplemental Figure 4) to determine if adipose leptin hypersecretion was contributing to the enhanced hyperleptinemia. Consistent with the systemic data, we demonstrated that both SC (IIH 8.3 ± 1.6 vs. controls 2.4 ± 0.4 ng/mL/100 mg; Welch’s *t* test; *t*_11.47_ = 3.6; *P* = 0.0039, Figure 5A) and OM (IIH 2.9 ± 0.8 vs. controls 0.6 ± 0.1 ng/mL/100 mg; Welch’s *t* test; *t*_9.276_ = 2.917; *P* = 0.016, Figure 5B) adipose tissue from IIH patients secreted more leptin compared with BMI-matched controls. Although adipose leptin secretion was elevated in IIH, this was independent of gene expression (Ob gene [leptin] from RNA sequencing) (Figure 5C) and adipocyte size (Figure 4).

### IIH adipose tissue is transcriptionally primed for lipid accumulation.

**Figure 6:**
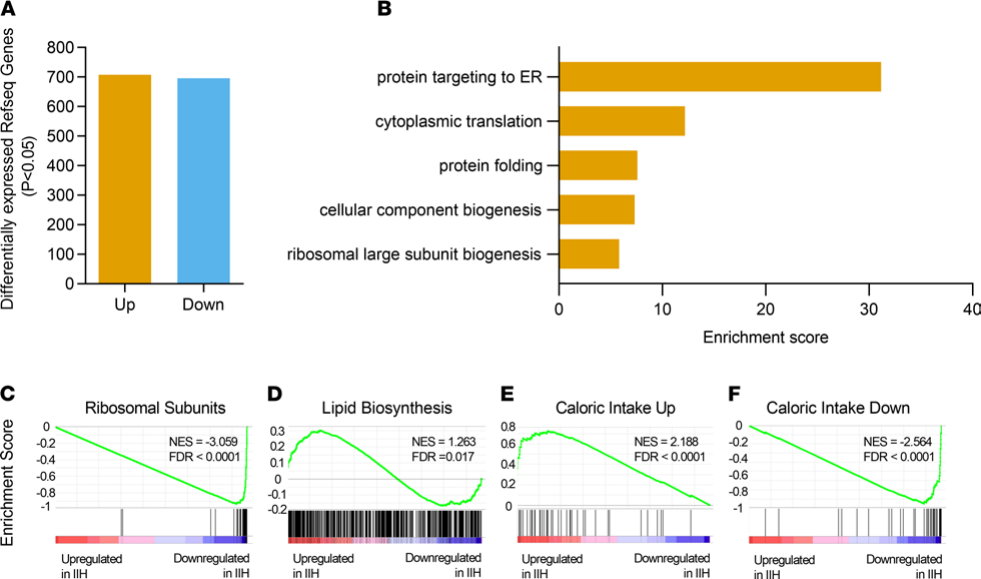
IIH SC adipose tissue displays a distinct transcriptome. Differential gene expression analysis of SC adipose tissue from control versus IIH patients. (**A**) Bar plot displaying the number of differentially expressed RefSeq genes at *P* < 0.05. (**B**) Gene ontology for significantly downregulated genes in IIH adipose. Gene set enrichment analysis of (**C**) Ribosomal Subunits, (**D**) Lipid Biosynthesis, (**E**) Caloric Intake Up, and (**F**) Caloric Intake Down against differential expression data from adipose tissue of control versus IIH patients. The green line represents the accumulation of genes in the indicated gene list against the expression pattern in control versus IIH patients (blue, downregulated in samples from IIH; red, upregulated in samples from IIH patients). Control *n* = 7, IIH *n* = 13. NES, normalized enrichment score; FDR, false discovery rate.

We examined the transcriptional profile of SC adipose tissue in patients with IIH compared with a control cohort matched for sex and BMI (Supplemental Figure 4). RNA sequencing followed by differential gene expression analysis revealed 708 upregulated and 696 downregulated National Center for Biotechnology Information (NCBI) RefSeq genes in IIH SC adipose tissue, based on *P* < 0.05 (Figure 6A); the genes can be found in Supplemental Data 1.

In order to identify signatures unique to IIH SC tissue, we performed gene ontology analysis using Database for Annotation, Visualization and Integrated Discovery (Figure 6B); gene lists and statistics can be found in Supplemental Data 2. Our analysis did not reveal gene pathways enriched in upregulated genes but revealed gene pathways prominently enriched in downregulated genes. Our results suggested that the enrichment for these pathways was mainly driven by the strongly downregulated ribosomal genes. Previous reports have linked suppression of the highly expressed ribosomal DNA gene transcription (ribosomal genes) as a prerequisite to lipid accumulation and energy storage (30–32). Therefore, we specifically interrogated ribosomal subunit genes (33), highly expressed genes, as well as gene sets regulated by caloric intake (34) and lipid biosynthesis for enrichment in IIH SC tissue using gene set enrichment analysis (GSEA) gene ontology analysis (Figure 6, C and D). We found that in IIH SC tissue, ribosomal genes were repressed, suggesting active energy storage (Figure 6C). Moreover, IIH SC transcriptional profile was enriched for gene expression changes associated with caloric intake during lipid biosynthesis (Figure 6, E and F). Both IIH SC and control SC tissues were derived from patients who had undergone an overnight fasting (routine before surgery); thus, gene expression profile in IIH SC tissue consistent with caloric intake and active lipidogenesis was unexpected. Taken together, SC adipose tissue in patients with IIH displayed a transcriptional profile consistent with active lipogenesis, despite the lack of caloric intake, suggesting an uncoupling of lipidogenesis from actual caloric intake.

### IIH adipocytes have perturbed metabolism.

Given our findings of elevated levels of insulin resistance in IIH and the demonstration that genes associated with lipogenesis were upregulated in SC adipose tissue (Figure 6), we conducted nontargeted nuclear magnetic resonance (NMR) metabolomics to provide insight into the metabolism of the IIH tissue compared with matched controls (Supplemental Figure 4). NMR spectra analysis allowed identification of 29 metabolites in explant cultured SC and OM adipose tissue (Supplemental Table 1) and 34 metabolites in the respective culture media (Supplemental Table 2).

**Figure 7:**
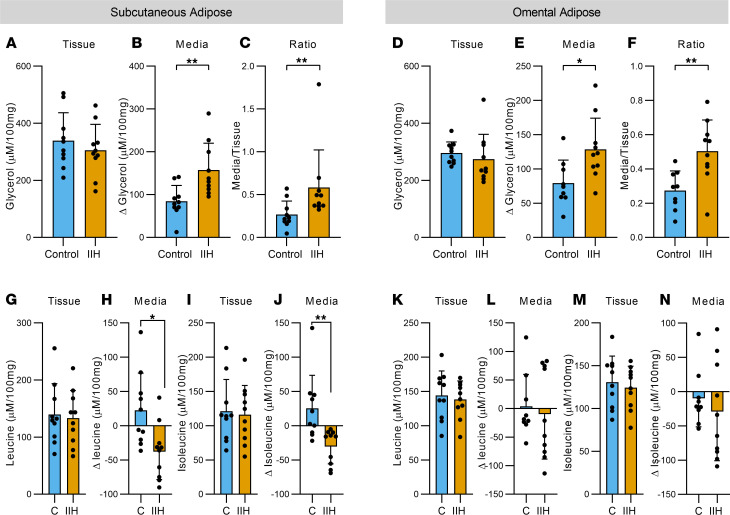
IIH adipose tissue displays features of altered lipid metabolism. NMR-based metabolomics on paired SC and OM adipose tissue explants and corresponding media in control and IIH patients. Tissue and media levels of glycerol in SC (*n* = 10) (**A**–**C**) and OM adipose tissue (control *n* = 9, IIH *n* = 10) (**D**–**F**). Tissue and media levels of leucine and isoleucine in SC (**G**–**J**) and OM (**K**–**N**) adipose tissue, *n* = 10. *n* represents a single patient’s adipose explant or corresponding media. Statistical tests: *t* tests for **A**, **B**, **E**–**G**, and **I**–**N** and Mann-Whitney *U* tests for **C**, **D**, and **H**. Data presented as mean ± SD; *P < 0.05, **P < 0.01.

IIH SC adipose tissue secreted more glycerol compared with controls (inferred from media incubated with adipose tissue relative to media without adipose tissue added, IIH 157.3 ± 62.6 vs. controls 84.5 ± 37.0 Δ μM/100 mg; unpaired 2-tailed *t* test, *t*_18_ = 3.168, *P* = 0.0053, Figure 7B). There was no difference in intracellular glycerol (Figure 7A), and therefore an increase in the secreted/intracellular glycerol ratio (0.58 ± 0.43 vs. 0.27 ± 0.16; Mann-Whitney *U* test, *U* = 14, *P* = 0.0052, Figure 7C), an indication of enhanced glycerol secretion. Similarly, IIH OM adipose tissue secreted more glycerol compared with controls (128.7 ± 45.7 vs. 79.4 ± 33.6 Δ μM/100 mg; unpaired *t* test, *t*_17_ = 2.655, *P* = 0.018, Figure 7E), without any alteration in intracellular glycerol (Figure 7D) and an increase in secreted/intracellular glycerol ratio (0.50 ± 0.18 vs. 0.27 ± 0.11; unpaired 2-tailed *t* test, *t*_17_ = 3.243, *P* = 0.0045, Figure 7F).

We detected alterations in branched-chain amino acid (BCAA) consumption, where the BCAAs, leucine and isoleucine, may be preferentially catabolized to lipogenic acetyl-CoA as previously shown to occur in adipocytes (35, 36). IIH SC adipose tissue displayed net uptake of both leucine (inferred from media incubated with adipose tissue relative to media without adipose tissue added, IIH –38.02 ± 40.76 vs. control 22.62 ± 54.25 Δ μM/100 mg; *t* test, *t*_18_ = 2.826, *P* = 0.011, Figure 7H) and isoleucine (IIH –30.22 ± 25.21 vs. controls 25.31 ± 48.26 Δ μM/100 mg; Mann-Whitney *U* test, *U* = 12, *P* = 0.002, Figure 7J), although adipose intracellular isoleucine and leucine levels were not altered (Figure 7, G and I). We suggest that IIH SC adipose tissue could be catabolizing these amino acids to support de novo lipogenesis. Conversely, IIH OM adipose showed no difference in uptake or intracellular isoleucine and leucine (Figure 7, K–N).

**Figure 8:**
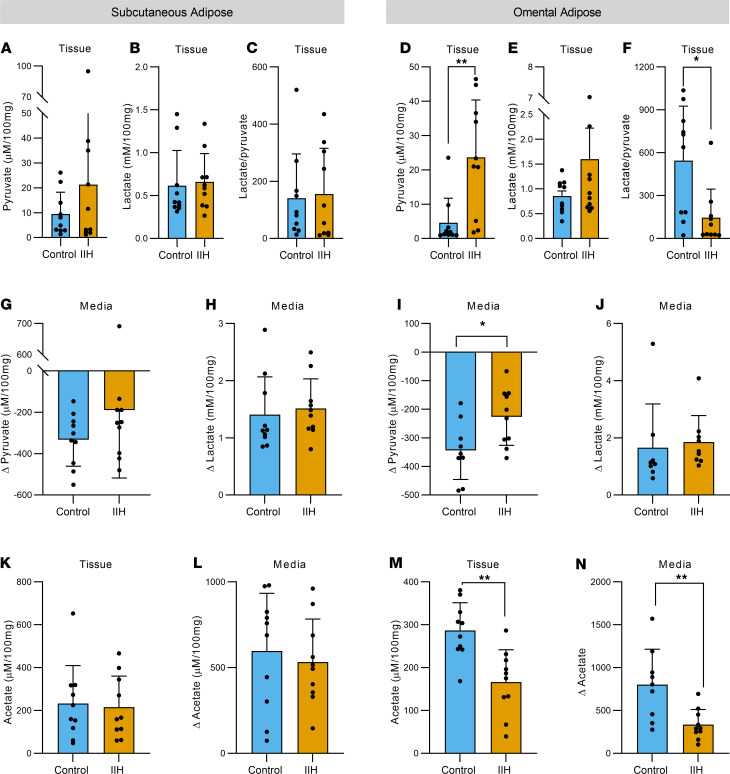
IIH OM adipose tissue displays features of altered nutrient utilization. NMR-based metabolomics on paired SC and OM adipose tissue explants and corresponding media in control and IIH patients. Tissue concentrations of pyruvate and lactate and pyruvate/lactate ratio in SC (**A**–**C**) and OM (**D**–**F**) adipose tissue. Media exchange of pyruvate and lactate in SC (**G** and **H**) and OM (**I** and **J**) adipose tissue. Tissue concentration and media exchange of acetate in SC (**K** and **L**) and OM (**M** and **N**) adipose tissue. *n* represents a single patient’s adipose explant or corresponding media. Statistical tests: *t* tests for **A**–**C** and **H**–**N** and Mann-Whitney *U* tests for **D**–**G**. Data presented as mean ± SD; *P < 0.05, **P < 0.01.

IIH OM adipose tissue had elevated tissue pyruvate (23.63 ± 16.77 vs. 4.58 ± 7.18 μM/100 mg; Mann-Whitney *U* test, *U* = 13, *P* = 0.0039, Figure 8D) with no change in tissue lactate (Figure 8E) and thus a decreased lactate to pyruvate ratio (145.0 ± 200 vs. 544.9 ± 380.7; Mann-Whitney *U* test, *U* = 18, *P* = 0.015, Figure 8F). We also showed reduced uptake of pyruvate into the OM adipose tissue (–226.1 ± 100.0 vs. –344.0 ± 101.7 Δ μM/100 mg; *t* test, *t*_17_ = 2.545, *P* = 0.0209, Figure 8I), suggestive of reduced tissue pyruvate consumption. No difference in lactate secretion was detected between the groups. In parallel, a reduction in tissue acetate was observed in the IIH OM adipose tissue alongside reduced secretion in the same depot (166.5 ± 75.22 vs. 286.8 ± 64.85 μM/100 mg; *t* test, *t*_18_ = 3.829, *P* = 0.0012, Figure 8M; and 335.4 ± 176.4 vs. 800.9 ± 414.6 Δ μM/100 mg; *t* test, *t*_17_ = 3.247, Figure 8N). No differences were observed in the SC adipose depot (Figure 8, A–C, G, H, and J–L).

## Discussion

IIH is a disease of elevated ICP predominantly among young women with obesity, where incidence is rising in line with global obesity trends. Epidemiological data have highlighted an increased risk of CVD in IIH in excess of that for obesity (6, 11). IIH has classically been regarded as a neuro-ophthalmic disease manifesting with headaches and risk of visual loss. Here, we provide what may be the first evidence that IIH is a disease of systemic metabolic dysregulation with neuro-ophthalmic manifestations. The insulin resistance phenotype in IIH is congruent with the previously described androgen excess phenotype in IIH, where female androgen excess is linked to insulin resistance and T2D (14). Although our relatively young IIH cohort (mean age 32) does not meet the criteria for prediabetes, the presence of insulin resistance in 50% of the cohort as assessed by HOMA2-IR, coupled with altered β cell function (HOMA-B) indicates a risk for progression to prediabetes and T2D, particularly in later life. This has important clinical implications for patient care, as insulin resistance is a potentially modifiable risk factor for future cardiometabolic morbidity (37).

Patients with IIH have hyperleptinemia in excess of obese controls and endorsed by elevated adipose leptin secretion. Previous studies also demonstrated raised serum leptin in IIH, where leptin was proposed to be causative in raised ICP (21). However, the lack of elevated CSF leptin and no correlation between leptin and LP OP suggest that hyperleptinemia is unlikely to be directly driving disordered CSF secretion in patients with IIH. Rather, we suggest that hyperleptinemia is a feature of systemic metabolic perturbation. Hyperleptinemia is a feature of other metabolic conditions and is associated with systemic insulin resistance, where insulin is a known leptin secretagogue (38, 39).

The relationship of the systemic metabolic perturbations in IIH to ICP dynamics and disease activity remains unclear. This study did not evaluate if the metabolic dysfunction was driving raised ICP, and this requires further investigation. The metabolic phenotype in IIH does, however, provide compelling evidence that IIH is not merely a disease of the CNS and eyes but a systemic metabolic disease. These metabolic features may be relevant to the previously documented, heightened CVD risk in IIH (6).

Obesity is a feature of metabolic disease, associated with insulin resistance and hyperleptinemia (40). Supporting the hypothesis that IIH is a disorder of systemic metabolic dysregulation, we noted increased truncal adiposity, which correlates with ICP, insulin resistance, and hyperleptinemia. Crucially, we show that patients with IIH have a greater proportion of truncal fat to lean mass, where excess fat mass is associated with insulin resistance (41).

Truncal adiposity correlated with LP OP, a marker of IIH disease activity; this suggests that adiposity could be associated with disease activity. In support of this, previous studies have described reduction of truncal fat mass in association with disease remission (8, 9). It is, however, unknown if weight loss confers improvement of the metabolic phenotype in IIH. We were able to access a cohort of IIH SC and IIH OM tissue despite its scarcity, providing the invaluable opportunity to perform in-depth analysis. We identified that IIH SC adipose tissue displayed a transcriptional profile consistent with active lipid biosynthesis following calorie intake, notable as both patients and controls were fasted at the time of the biopsy (30, 34). As such, these findings suggest that SC adipose tissue is geared for lipogenesis. We noted downregulation of highly expressed ribosomal genes. This is in keeping with previous literature demonstrating that during active lipogenesis, adipose tissue downregulates the transcription of highly expressed genes (such as ribosomal genes) (34). Our metabolomic data suggest IIH adipose metabolism was dysregulated compared with control obese adipose tissue. IIH SC adipose tissue showed increased capacity for uptake of BCAAs, where isoleucine and leucine catabolism could contribute up to a quarter of the lipogenic acetyl-CoA pool (35). These data support that IIH SC adipose tissue can preferentially catabolize BCAAs to support increased lipogenesis, corroborating the transcriptomic data (34). Given the indicators of increased lipogenesis, the elevated glycerol secretion from IIH adipocytes is unlikely to be derived from lipolysis. It is therefore possible that in keeping with previous studies, and the insulin-resistant phenotype we have noted in IIH, the increased glycerol secretion could reflect breakdown of excess glucose through glyceroneogenesis within the SC adipose tissue (42).

Together these data could indicate that patients with IIH are predisposed to gaining adipose mass, which is important to consider when IIH patients often experience an exacerbation or onset of symptoms following rapid weight gain (7, 43). Dynamic assessment of de novo lipogenesis in IIH SC adipose tissue would help support this hypothesis.

In OM adipose tissue we identified decreased pyruvate uptake coupled with increased tissue levels of pyruvate. This is in the context of unchanged tissue and media lactate levels, suggesting this is not occurring because of sensitivity to hypoxia but more likely a means of the cell maintaining favorable cytosolic redox homeostasis under challenging metabolic conditions. This is, however, occurring in the setting of reduced tissue acetate and acetate secretion. Taken together our potentially novel tissue approach has revealed that in IIH the OM depot maintains a more efficient energy network while the SC depot shows more signs of metabolic dysfunction, potentially contributing to disease pathophysiology and cardiovascular risk.

The study findings are limited to adult women, rather than male (5% of IIH) or pediatric patients, with IIH (5). By nature of the disease being prevalent during childbearing years, the cohort is relatively young, and consequently further studies are now warranted to evaluate metabolic implications for a more aged IIH population, where metabolic complications may be more severe. Our studies have found evidence of metabolic dysfunction compared to obesity despite a young age compared with controls (44). The typical young age of patients with IIH is important as earlier intervention to modify cardiometabolic risk factors is likely to improve future mortality and morbidity from CVD as seen in other conditions characterized by metabolic dysfunction (45, 46). The present study utilized a relatively small sample size compared with other studies that assess more common diseases. Additionally, our adipose tissue studies were powered based on previous similar studies. However, we cannot eliminate the possibility of larger sample sizes yielding different results. However, the data were strengthened by the detailed clinical phenotyping, as well as the notable number of subjects with IIH, considering that IIH is a rare disease. The results lay the foundation for a prospective in-depth metabolic assessment across the IIH life course.

**Figure 9:**
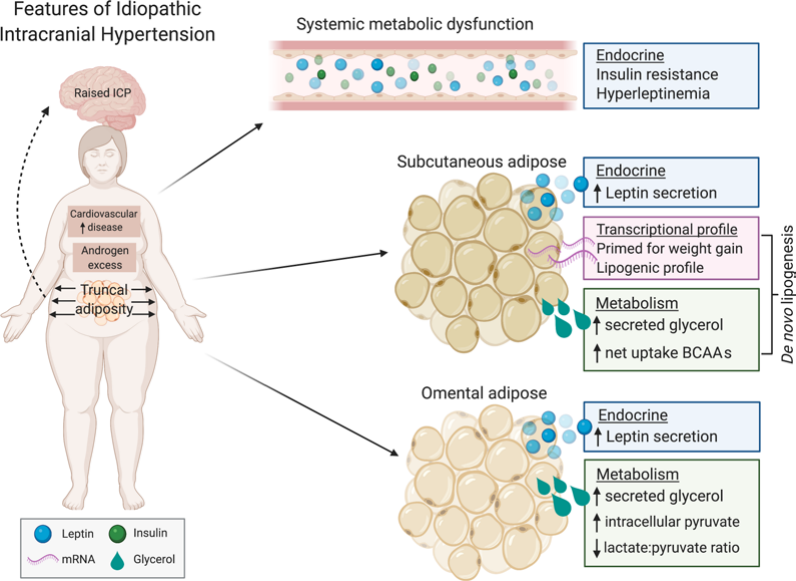
IIH metabolism concept figure. IIH patients display systemic and tissue-level metabolic disruption in excess of that conferred by obesity. IIH patients are insulin resistant and display hyperleptinemia, where they have increased abdominal obesity. IIH adipose tissue displays leptin hypersecretion and features of transcriptomic and metabolic dysfunction.

We provide what we believe to be the first description of detailed metabolic phenotyping in active IIH, defining contributions to cardiovascular risk and identifying adipose tissue mechanisms that may contribute to pathophysiology (Figure 9). Adiposity in IIH is preferentially truncal, with SC adipocytes demonstrating increased leptin secretion and transcriptional priming for caloric storage and gaining adipose mass. We also note differential fuel utilization in the OM adipose. The adipose phenotype described may be contributing to insulin resistance and will need further evaluation. These data indicate that IIH is likely a systemic metabolic disease with neuro-ophthalmic features rather than solely a neuro-ophthalmological disease. We have not determined the causal relationship between the metabolic derangement and ICP dysregulation in IIH, and this would be worthy of future investigation. The metabolic phenotype is likely to explain the increased risk of CVD and T2D in IIH. As IIH presents in early adulthood, modifying the metabolic aspects of the disease through addressing insulin resistance and managing cardiovascular risk factors could improve patient long-term outcomes.

## Methods

Unless otherwise stated, materials are from MilliporeSigma.

### Study design.

A case-control study comparing IIH with matched controls was conducted to assess the systemic metabolic profile: BMI, BP, fasting glucose and insulin, cholesterol and triglycerides, and leptin (serum and CSF). Substudy 1 evaluated the hepatic and renal profile, and Substudy 2 evaluated the body composition and distribution (Supplemental Figure 1). Adipose tissue was then evaluated in separate IIH and control populations.

### Study population.

Young (ages 16–55) female IIH patients with active IIH (papilledema > grade 1 Frisen and LP OP > 25 cmCSF on the date of research assessment visit) were recruited. The clinical consequences of IIH were not evaluated in this study; rather underling systemic disease activity was assessed. Hence, patients with IIH at any stage of disease were included in the present analysis given that they had active disease. Patients who had previously failed pharmacotherapy, were undergoing pharmacotherapy (such as acetazolamide), or had failed community weight management were included in the study given the presence of active IIH; thus, the IIH cohort represents a cohort with active disease. Control patients met the same inclusion criteria as the IIH patients, where absence of IIH was confirmed. The control subject cohort was matched to the IIH population for age, sex, and BMI. Sex was participant reported.

### Exclusion criteria.

Exclusion criteria for all patients included receiving hormone-manipulating medication, significant comorbidities including known endocrinopathies, and the inability to give informed consent. Additionally, patients with IIH were excluded if they were pregnant during the visit.

### Assessments.

All participants underwent detailed medical history and examination. All blood samples were collected following an overnight fast (from midnight). Lumbar punctures were carried out in all IIH patients and conducted in the left lateral decubitus with knees bent at a 90° angle or more and LP OP recorded before CSF was collected (up to 15 mL). Serum samples not analyzed immediately were centrifuged (10 minutes at 1500*g* at 4°C), aliquoted, and stored at –80°C. CSF samples were centrifuged (800*g* for 10 minutes at 4°C), and the supernatant was aliquoted and stored at –80°C. All samples processed only underwent a single freeze-thaw cycle.

### Clinical and biochemical analysis.

BMI was calculated from weight and height using the following formula: BMI = (weight [kg] / height [m]^2^). Fasting glucose, Hb1Ac, and lipids were measured. In Substudy 1 subjects also had liver function (bilirubin, ALP, and AST) and renal function tests (urea, creatinine, and eGFR, calculated using the Chronic Kidney Disease Epidemiology collaboration equation). All tests were conducted in the biochemistry department at University Hospital Birmingham NHS Foundation Trust, United Kingdom.

### Fasting insulin and HOMA2-IR.

Fasting insulin was measured using commercially available assays (Mercodia), according to the manufacturer’s instructions. HOMA2-IR was calculated using the program HOMA calculator v2.2.3 (https://www.dtu.ox.ac.uk/homacalculator/).

### Body composition.

DEXA was performed using a total-body scanner (QDR 4500; Hologic), as previously described (20, 47), on a subset of patients. The scans were conducted by a clinical scientist and trained radiographer. Patients with metal prosthetics or implants were included, and tissue overlying the prosthesis was excluded from analysis. Scans were checked for accuracy of fields of measurement. Regional fat mass was analyzed as described previously (20, 47). The precision of total fat mass measures in terms of coefficient of variation (CV) was less than 3%, and for regional fat analyses it was less than 5%. Both the IIH and control cohorts were analyzed on the same DEXA scanner.

Additionally, a subset of patients had body fat percentage determined by bio-impedance via a Body Composition Analyser (TANITA BC-418 MA). A 0.2 kg correction was made for base layer clothing; a standard female body type preset was selected for all patients. The machine was used according to manufacturer’s instructions.

### Adipose tissue collection.

Adipose tissue collection from patients with IIH was covered under ethical approvals (13/YH/0366 and 14/WM/0011). Bariatric control patients were identified from elective bariatric lists at Birmingham Heartlands Hospital NHS Trust who had no endocrinopathies and were not on hormonal treatments under the ethics approval (14/WM/0011). All patients were fasted overnight (from midnight) prior to adipose tissue biopsy. Adipose tissue (abdominal SC) and where possible OM was biopsied and was placed immediately in RNAlater, into phenol-free DMEM/F12 (Thermo Fisher Scientific) without antibiotics, or into 4% formaldehyde.

### Histomorphometric analysis.

Adipose tissue was fixed in 4% formaldehyde prior to dehydration, clearing, and embedding in paraffin wax. Embedded tissue was cut in 5 μm sections prior to an H&E stain. Sections were imaged using a Leica DM ILM inverted microscope (Leica Microsystems) though a Leica DFC290 camera (Leica Microsystems) utilizing the Leica application suite (V2.8.1, Leica Microsystems). Adipocyte area was assessed via the ImageJ (NIH) plugiin Adiposoft (48). The evaluator was blinded to tissue type and patient disease state during analysis.

### RNA sequencing.

Stranded mRNA cDNA libraries derived from SC adipose tissue (insufficient OM tissues precluded this analysis) were sequenced at 2X100 paired-end reads on the Illumina HiSeq 2500 platform by Eurofins Genomics. Control and IIH RNA had comparable RNA integrity number quality (7.5 ± 0.82 vs. 7.7 ± 0.50, *P* = 0.6), indicating suitable RNA integrity.

Quality control on the RNA sequencing was performed with FastQC v0.11.4. Read and adapter trimming was carried out using TrimGalore! v0.4.4 with Cutadept v1.13 with default settings (49). RNA-sequencing reads were mapped to the human genome (hg19, UCSC annotation) utilizing STAR software v2.5.3a with default parameters (50). Counts per gene were calculated using custom scripts acting in a HTSeqcount compatible mode with the following parameters: --format=bam --minaqual=10 --stranded=reverse –mode=union (51, 52). Differentially expressed genes were identified using DESeq2 (v1.14.1) from Bioconductor release 3.3 (53). Differentially expressed genes were called at an FDR of 5%. Normalized FPKM values for each gene were calculated using DESeq2 and GenomicFeatures v1.26.4 package (54). GSEA was carried out as described previously (55, 56). Interrogated gene sets can be found in Supplemental Data 3, where gene sets were derived from previous articles (30, 33, 34).

### Conditioned media protocol.

Adipose tissue had large blood vessels dissected out and was cut into approximately 100 mg explants prior to a 24-hour incubation in phenol-free DMEM/F12 with no antibiotics, in glass tubes (VWR) at 37°C. Following incubation, the media were aliquoted, and corresponding explants were stored at –80°C prior to analysis.

### Metabolomics.

NMR-based metabolomics provided a nontargeted metabolomics approach. Adipose tissue–conditioned media 1 in 4 in NMR buffer (final concentration: 100 mM sodium phosphate, 500 μM 4,4-dimethyl-4-silapentane-1-sulfonic acid [DSS], 2 mM imidazole, and 10% deuterium [D_2_O]). Corresponding SC and OM explants underwent a methanol/water/chlorophorm extraction prior to retention and evaporation of the polar layer. Dried samples were reconstituted in 60 μL of 100 mM sodium phosphate buffer containing 100% D_2_O and 500 μM DSS. All samples were transferred into 1.7 mm Bruker Sample Jet NMR tubes (Cortecnet) via an automatic Gilson.

Samples were run on a Bruker 600 MHz Bruker Avance III spectrometer (Bruker Biospin) with a TCl 1.7 mm z-PGF cryogenic probe set at 300 K. One-dimensional (1D) ^1^H-NMR spectra were obtained, where spectral width was set to 7812.5.

1D ^1^H-NMR spectra were processed using MetaboLab software (57). All 1D data sets were 0-filled to 131,072 data points prior to Fourier transformation. The chemical shift was calibrated by referencing the DSS signal to 0 parts per million. 1D spectra were manually phase corrected. Batch baseline correction was achieved using a spline function. 1D ^1^H-NMR spectra were exported into Bruker format for metabolite identification and concentration determination using Chenomx 8.2. All values obtained were normalized to the mass of the appropriate adipose tissue explant. Conditioned media values were made relative to media without adipose tissue. The investigator was blinded to patient type and tissue type during metabolite quantification.

### Leptin ELISAs.

Leptin was quantified in adipose conditioned medium, serum, and CSF using the human leptin DuoSet ELISA (DY-398, Bio-Techne). ELISA was carried out according to the manufacturer’s instructions using the recommended ancillary kit (Bio-Techne, DY008). Conditioned medium was diluted 1:50, serum 1:100, and CSF 1:5 in reagent diluent. Samples were run in duplicate. Total secreted leptin was normalized to corresponding explant mass. Intra-assay variability was CV 7.28 %, and inter-assay variability was CV 8.2% for conditioned medium assay. Serum intra-assay variability was CV 2.71 % and inter-assay variability was CV 6.99 %. CSF yielded intra-assay variability CV 3.85 % and inter-assay variability CV 8.9%.

### Data availability.

The accession code for RNA-sequencing data from the Gene Expression Omnibus database is GSE171398.

### Statistics.

Statistical analysis was performed using GraphPad Prism 8 (GraphPad Software Inc) and SPSS 24 (SPSS Inc). Data are presented as mean ± SD unless otherwise stated. Data normality was assessed by a Shapiro-Wilk normality test. Where data were normally distributed, unpaired 2-tailed *t* test (equal variance) or Welch’s test (unequal variance) was employed, whereas nonparametric data were assessed via Mann-Whitney *U*. Spearman’s rank correlation coefficient (*ρ*) and Pearson’s correlation coefficient (*r*) were used for assessing correlations in the IIH cohorts. Where data points are missing, data were not imputed. We did not correct for multiple comparisons because this would have increased the likelihood of type II errors with the exception of RNA-sequencing data. Results were judged significant at *P* < 0.05.

### Study approval.

IIH subjects were identified from multiple UK centers, and samples were collected following receipt of informed, written consent. The trials received ethical approval from the Yorkshire and the Humber–Leeds West Research Ethics Committee (REC) (13/YH/0366, Leeds, United Kingdom), Dudley local REC (06/Q2702/64, Dudley, United Kingdom), and the Black Country REC (14/WM/0011, Dudley, United Kingdom).

Control patients were recruited via advertisement; sample collection occurred following informed, written consent. Sample collection was approved by the South Birmingham Local REC (Birmingham, United Kingdom) and the Black Country REC (14/WM/0011). Control patients for adipose tissue experiments were recruited from NHS elective bariatric surgery lists following written informed consent, under protocols approved by the Black Country REC (14/WM/0011).

## Author contributions

AJS designed and conceived the study. CSJW, HFB, ZA, KAM, AY, JLM, and CL conducted the experiments. AY, JLM, KAM, and AJS conducted clinical assessments. CSJW, AY, RS, JLM, KAM, and AJS collected clinical samples. CSJW, HFB, ZA, MW, GS, DH, and IA analyzed the data. CSJW, HFB, IA, SPM, GGL, and AJS drafted the manuscript. CSJW, HFB, ZA, AY, MW, GS, RS, JLM, OG, KAM, DH, DAT, JWT, SPM, CL, IA, GGL, AJS read the manuscript for intellectual content and approved the final version of the paper.

## Supplementary Material

Supplemental data

Trial reporting checklists

ICMJE disclosure forms

Supplemental Data Set 1

Supplemental Data Set 2

Supplemental Data Set 3

## Figures and Tables

**Table 1:**
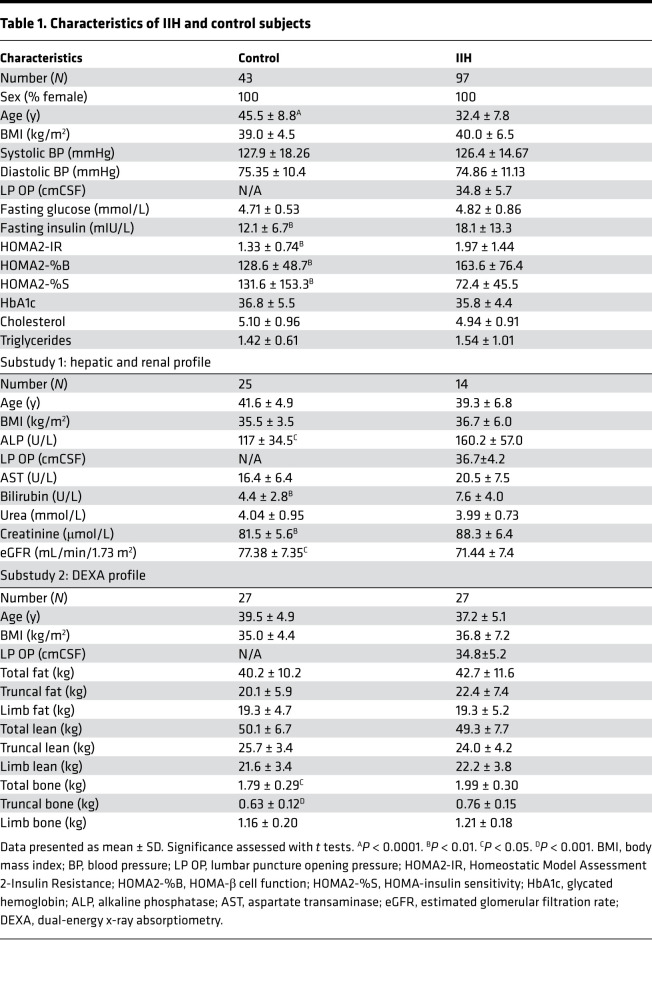
Characteristics of IIH and control subjects

## Version 1 04/13/2021

In-Press Preview

## Version 2 05/24/2021

Electronic publication

## References

[1] Mulla Y (2015). Headache determines quality of life in idiopathic intracranial hypertension. J Headache Pain.

[2] Mollan SP (2019). Advances in the understanding of headache in idiopathic intracranial hypertension. Curr Opin Neurol.

[3] Mollan SP (2018). Idiopathic intracranial hypertension: consensus guidelines on management. J Neurol Neurosurg Psychiatry.

[4] Corbett JJ (1982). Visual loss in pseudotumor cerebri. Follow-up of 57 patients from five to 41 years and a profile of 14 patients with permanent severe visual loss. Arch Neurol.

[5] Mollan SP (2018). The expanding burden of idiopathic intracranial hypertension. Eye (Lond).

[6] Adderley NJ (2019). Association between idiopathic intracranial hypertension and risk of cardiovascular diseases in women in the United Kingdom. JAMA Neurol.

[7] Giuseffi V (1991). Symptoms and disease associations in idiopathic intracranial hypertension (pseudotumor cerebri): a case-control study. Neurology.

[8] Hornby C (2018). Evaluating the fat distribution in idiopathic intracranial hypertension using dual-energy X-ray absorptiometry scanning. Neuroophthalmology.

[9] Sinclair AJ (2010). Low energy diet and intracranial pressure in women with idiopathic intracranial hypertension: prospective cohort study. BMJ.

[10] Mollan SP (2019). What are the research priorities for idiopathic intracranial hypertension? A priority setting partnership between patients and healthcare professionals. BMJ Open.

[11] Frič R (2017). Cardiovascular risk factors in Chiari malformation and idiopathic intracranial hypertension. Brain Behav.

[12] Mani H (2013). Diabetes and cardiovascular events in women with polycystic ovary syndrome: a 20-year retrospective cohort study. Clin Endocrinol (Oxf).

[13] O’Reilly MW (2019). A unique androgen excess signature in idiopathic intracranial hypertension is linked to cerebrospinal fluid dynamics. JCI Insight.

[14] O’Reilly MW (2019). Serum testosterone, sex hormone-binding globulin and sex-specific risk of incident type 2 diabetes in a retrospective primary care cohort. Clin Endocrinol (Oxf).

[15] Tabák AG (2009). Trajectories of glycaemia, insulin sensitivity, and insulin secretion before diagnosis of type 2 diabetes: an analysis from the Whitehall II study. Lancet.

[16] Geloneze B (2009). HOMA1-IR and HOMA2-IR indexes in identifying insulin resistance and metabolic syndrome: Brazilian Metabolic Syndrome Study (BRAMS). Arq Bras Endocrinol Metabol.

[17] Anderson KM (1991). Cardiovascular disease risk profiles. Am Heart J.

[18] Fujioka S (1987). Contribution of intra-abdominal fat accumulation to the impairment of glucose and lipid metabolism in human obesity. Metabolism.

[19] Peiris AN (1989). Adiposity, fat distribution, and cardiovascular risk. Ann Intern Med.

[20] Tomlinson JW (2008). Impaired glucose tolerance and insulin resistance are associated with increased adipose 11beta-hydroxysteroid dehydrogenase type 1 expression and elevated hepatic 5alpha-reductase activity. Diabetes.

[21] Ball AK (2009). Elevated cerebrospinal fluid (CSF) leptin in idiopathic intracranial hypertension (IIH): evidence for hypothalamic leptin resistance?. Clin Endocrinol (Oxf).

[22] Hornby C (2018). Metabolic concepts in idiopathic intracranial hypertension and their potential for therapeutic intervention. J Neuroophthalmol.

[23] Lampl Y (2002). Serum leptin level in women with idiopathic intracranial hypertension. J Neurol Neurosurg Psychiatry.

[24] Dhungana S (2009). Cytokines and chemokines in idiopathic intracranial hypertension. Headache.

[25] Behbehani R (2010). Is cerebrospinal fluid leptin altered in idiopathic intracranial hypertension?. Clin Endocrinol (Oxf).

[26] Samancl B (2017). Evidence for potential involvement of pro-inflammatory adipokines in the pathogenesis of idiopathic intracranial hypertension. Cephalalgia.

[27] Lönnqvist F (1997). Leptin secretion from adipose tissue in women. Relationship to plasma levels and gene expression. J Clin Invest.

[28] Schwartz MW (1996). Cerebrospinal fluid leptin levels: relationship to plasma levels and to adiposity in humans. Nat Med.

[29] Amitani M (2013). Front Neurosci.

[30] Oie S (2014). Hepatic rRNA transcription regulates high-fat-diet-induced obesity. Cell Rep.

[31] Murayama A (2008). Epigenetic control of rDNA loci in response to intracellular energy status. Cell.

[32] Grummt I, Ladurner AG (2008). A metabolic throttle regulates the epigenetic state of rDNA. Cell.

[33] Yoshihama M (2002). The human ribosomal protein genes: sequencing and comparative analysis of 73 genes. Genome Res.

[34] Franck N (2011). Identification of adipocyte genes regulated by caloric intake. J Clin Endocrinol Metab.

[35] Crown SB (2015). Catabolism of branched chain amino acids contributes significantly to synthesis of odd-chain and even-chain fatty acids in 3T3-L1 adipocytes. PLoS One.

[36] Rosenthal J (1974). Metabolic fate of leucine: a significant sterol precursor in adipose tissue and muscle. Am J Physiol Content.

[37] Tabák AG (2012). Prediabetes: a high-risk state for diabetes development. Lancet.

[38] Malmström R (1996). Insulin increases plasma leptin concentrations in normal subjects and patients with NIDDM. Diabetologia.

[39] Segal KR (1996). Relationship between insulin sensitivity and plasma leptin concentration in lean and obese men. Diabetes.

[40] Després JP, Lemieux I (2006). Abdominal obesity and metabolic syndrome. Nature.

[41] Ezeh U (2014). Association of fat to lean mass ratio with metabolic dysfunction in women with polycystic ovary syndrome. Hum Reprod.

[42] Rotondo F (2017). Glycerol is synthesized and secreted by adipocytes to dispose of excess glucose, via glycerogenesis and increased acyl-glycerol turnover. Sci Rep.

[43] Andrews LE (2014). Idiopathic intracranial hypertension and obesity. Horm Res Paediatr.

[44] Ferrannini E (1996). Insulin action and age. European Group for the Study of Insulin Resistance (EGIR). Diabetes.

[45] Herman WH (2015). Early detection and treatment of type 2 diabetes reduce cardiovascular morbidity and mortality: a simulation of the results of the Anglo-Danish-Dutch Study of intensive treatment in people with screen-detected diabetes in primary care (ADDITION-Europe). Diabetes Care.

[46] Feldman AL (2017). Screening for type 2 diabetes: do screen-detected cases fare better?. Diabetologia.

[47] Stewart PM (1999). Cortisol metabolism in human obesity: impaired cortisone-- *>*cortisol conversion in subjects with central adiposity. J Clin Endocrinol Metab.

[48] Galarraga M (2012). Adiposoft: automated software for the analysis of white adipose tissue cellularity in histological sections. J Lipid Res.

[49] Martin M (2011). Cutadapt removes adapter sequences from high-throughput sequencing reads. EMBnet J.

[50] Dobin A (2013). STAR: ultrafast universal RNA-seq aligner. Bioinformatics.

[51] Anders S (2015). HTSeq--a Python framework to work with high-throughput sequencing data. Bioinformatics.

[52] Dyer NP (2019). LiBiNorm: an htseq-count analogue with improved normalisation of Smart-seq2 data and library preparation diagnostics. PeerJ.

[53] Love MI (2014). Moderated estimation of fold change and dispersion for RNA-seq data with DESeq2. Genome Biol.

[54] Lawrence M (2013). Software for computing and annotating genomic ranges. PLoS Comput Biol.

[55] Akerman I (2017). Human pancreatic β cell lncrnas control cell-specific regulatory networks. Cell Metab.

[56] Elhassan YS (2019). Nicotinamide riboside augments the aged human skeletal muscle NAD^+^ metabolome and induces transcriptomic and anti-inflammatory signatures. Cell Rep.

[57] Ludwig C, Gunther UL (2011). MetaboLab--advanced NMR data processing and analysis for metabolomics. BMC Bioinformatics.

