# Supplementary material for: Systemic and adipocyte transcriptional and metabolic dysregulation in idiopathic intracranial hypertension
Source: JCI Insight. 2021 May 24;6(10):e145346. doi: 10.1172/jci.insight.145346 (PMC8262372; doi:10.1172/jci.insight.145346)
Supplement: Supplemental data [file jciinsight-6-145346-s011.pdf]

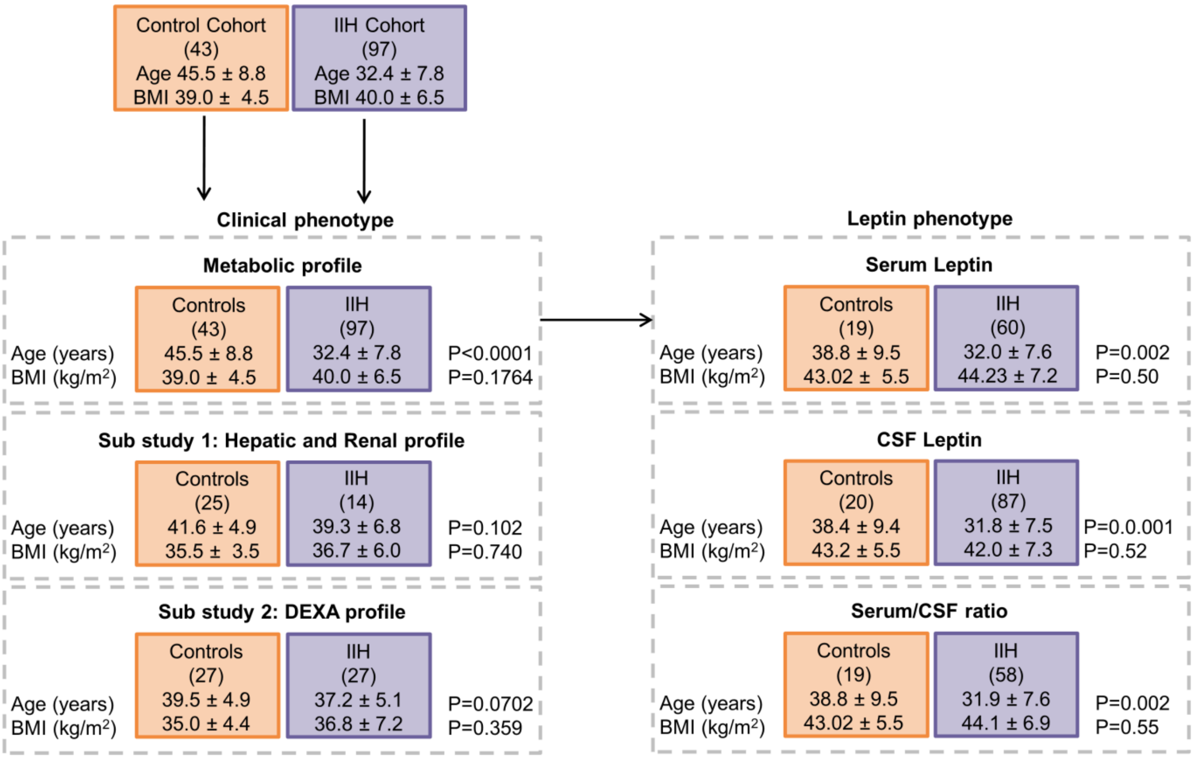


**Figure S1 Consort diagram**

Demographic characteristics for the control and IIH patients within the study and the sub-studies. Numbers in parentheses represent N for respective group. Data presented as mean±standard deviation. BMI= Body mass index.

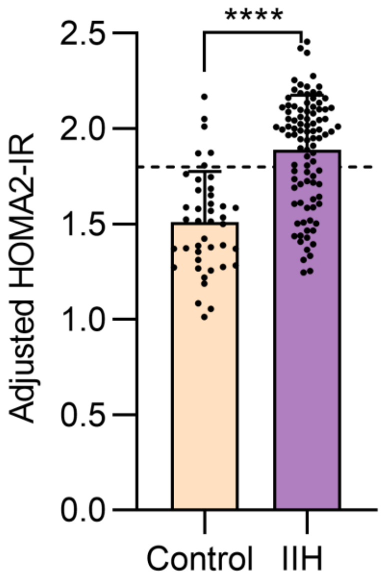


**Figure S2) HOMA2-IR**

Histograms of fasted HOMA2-IR adjusted for age and BMI via multiple regression analysis in control (n=43) and IIH patients (n=97). Dotted line represents threshold suggestive of type 2 diabetes mellitus.. N represents an individual patient. Data presented as mean±SD, ****=P<0.0001.

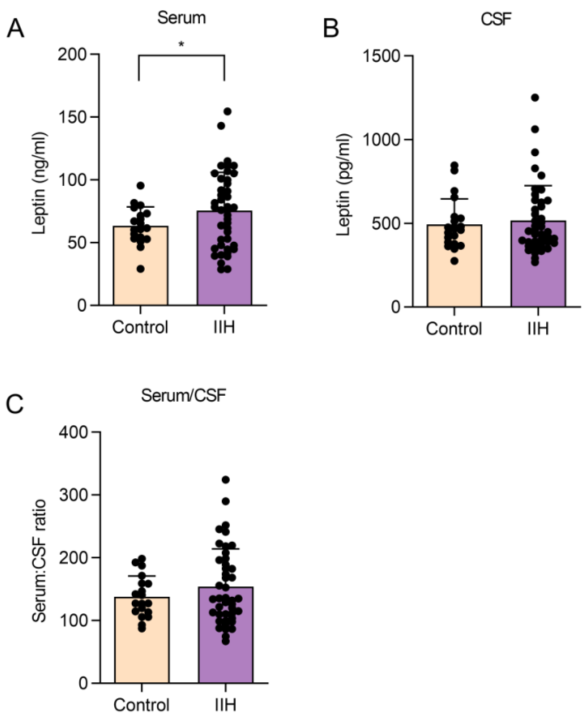


**Figure S3) Leptin sensitivity analysis, relating to figure 3**

A sex, BMI and age matched cohort of control and IIH patients were sub analysed for (**A**) serum, (**B**) CSF leptin and (**C**) serum/CSF leptin ratio. (D) Table of patient variables. Welch's for A, Mann-Whitney test for B and C. N=19 for control, N=43 for IIH. Data presented as mean±SD, *=P<0.05.

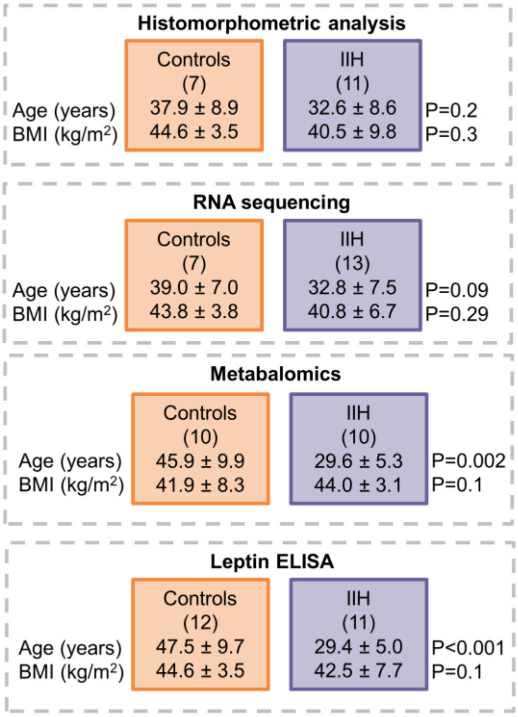


**Figure S4) Adipose tissue group matching**

Patient characteristics for adipose tissue experiments. Numbers in parentheses represent N for respective group. Data presented as mean±SD and T-tests.

| | Subcutaneous | | | Omental | | |
|---|---|---|---|---|---|---|
| µM/100mg | Control | IIH | P | Control | IIH | P |
| Acetate | 232.7(176.8) | 215.4(145.5) | 0.81 | 286.8 (64.8) | 166.5 (75.2) | 0.0012* |
| Alanine | 51.1 (20.8) | 82.1 (43.0) | 0.06 | 62.6 (28.7) | 109.0 (73.4) | *0.052* |
| Arginine | 202.8 (62.4) | 215.8(117.7) | 0.76 | 246.9 (72.2) | 238.3 (88.4) | 0.81 |
| Formate | 57.6 (47.9) | 72.5 (52.9) | *0.35* | 56.1 (28.5) | 53.9 (34.7) | *0.73* |
| Glucose (mM) | 3.7 (1.6) | 3.6 (1.2) | 0.86 | 4.1 (1.1) | 3.6 (1.5) | 0.43 |
| Glutamate | N/A | | | 146.5 (63.0) | 150.6 (69.0) | *0.79* |
| Glutamine | 551.5(287.2) | 509.7(253.5) | 0.73 | 679.6(241.7) | 719.6(233.7) | 0.71 |
| Glutathione | 13.2 (6.6) | 22.9 (17.6) | *0.12* | 20.9 (13.3) | 16.7 (5.6) | *0.57* |
| Glycerol | 339.0 (97.9) | 305.7 (90.8) | 0.44 | 296.1 (38.5) | 274.1 (87.4) | *0.16* |
| Glycine | 105.5 (44.6) | 117.3 (47.7) | 0.57 | 132.8 (36.3) | 148.5 (68.2) | 0.53 |
| *Histidine* | 41.9 (21.3) | 36.4 (13.1) | 0.49 | 38.9 (10.3) | 43.5 (10.0) | 0.32 |
| *Isoleucine* | 121.0 (46.6) | 116.1 (42.8) | 0.81 | 130.9 (30.3) | 124.4 (25.0) | 0.6 |
| Lactate (mM) | 616.2(409.3) | 660.5(330.1) | *0.48* | 0.85 (0.33) | 1.59 (1.97) | *0.48* |
| *Leucine* | 139.8 (53.8) | 133.3 (48.3) | 0.78 | 144.3 (35.6) | 138.3 (26.9) | 0.67 |
| Lysine | 134.1 (60.4) | 151.1 (55.8) | 0.52 | 162.1 (51.10 | 172.1 (43.0) | 0.64 |
| *Methionine* | 34.1 (17.5) | 29.7 (17.6) | 0.58 | 34.1 (9.4) | 41.3 (11.2) | 0.13 |
| Methylsuccinate | 5.1 (3.7) | 4.3 (2.4) | *0.85* | 4.9 (2.8) | 7.1 (5.1) | *0.49* |
| *Phenylalanine* | 60.0 (27.4) | 58.5 (19.7) | 0.89 | 65.1 (18.7) | 72.5 (15.7) | 0.34 |
| Proline | 56.1 (38.2) | 60.1 (30.7) | 0.39 | 74.5 (24.9) | 83.1 (23.4) | 0.43 |
| Pyroglutamate | 191.6 (89.3) | 184.2(132.1) | 0.88 | 190.0 (84.4) | 181.9 (86.3) | 0.83 |
| Pyruvate | 9.5 (8.7) | 21.3 (29.4) | *0.79* | 4.5 (7.1) | 23.6 (16.7) | *0.003** |
| Sarcosine | 6.7 (2.6) | 6.4 (2.9) | 0.81 | 5.4 (1.4) | 6.7 (2.6) | 0.22 |
| Succinate | N/A | | | 13.5 (4.6) | 18.2 (8.4) | 0.14 |
| Threonine | 149.4 (57.7) | 166.9 (70.0) | *0.48* | 188.3 (44.5) | 196.1 (60.4) | 0.74 |
| Tryptophan | 10.6 (4.6) | 11.3 (2.9) | 0.66 | 12.3 (3.5) | 13.8 (3.4) | 0.34 |
| Tyrosine | 71.4 (30.4) | 75.9 (28.7) | 0.74 | 87.4 (23.4) | 95.0 (21.4) | 0.46 |
| *Valine* | 144.6 (52.5) | 142.3 (50.9) | 0.92 | 159.3 (38.3) | 157.1 (31.1) | 0.89 |
| myo-Inositol | 162.0 (74.1) | 171.4 (86.7) | 0.79 | 130.7 (59.0) | 147.2 (86.7) | *0.91* |
| Sn-glycero-3-PC | 94.0 (26.9) | 95.9 (54.8) | 0.79 | 83.9 (27.5) | 77.3 (22.6) | 0.56 |

| Ratios | Control | IIH | P | Control | IIH | P |
|---|---|---|---|---|---|---|
| Lactate/Pyruvate | 140.6(154.9) | 155.2(160.3) | *0.85* | 544.9(380.7) | 145.0(199.9) | *0.015** |

**Table S1) Adipose tissue metabolite concentration**

Concentration of tissue explant metabolite concentration in adipose explants from paired OM and SC adipose tissue from IIH and control patients as determined by NMR. Statistical significance is denoted by *, where p<0.05 is considered significant. Numbers marked in *Italics*= Mann-Whitney U-test, unmarked = unpaired T-test. Data presented as mean±SD.

| | Subcutaneous | | | Omental | | |
|---|---|---|---|---|---|---|
| ΔμM/100mg | Control | IIH | P | Control | IIH | P |
| Acetate | 596.6 (336.8) | 532.5 (250.5) | 0.63 | 800.9(414.6) | 335.4(176.4) | 0.004* |
| Adenosine | -2.8 (0.4) | -1.9 (0.8) | 0.012* | N/A | | |
| Alanine | 87.1 (84.8) | 141.6 (84.3) | 0.16 | 67.9 (43.9) | 102.8(53.4) | 0.14 |
| Arabinose | -32.2 (5.2) | -23.1 (22.9) | *0.23* | N/A | | |
| Arginine | -70.9 (90.9) | -90.4 (90.6) | *0.31* | -227.0 (274.7) | -58.4 (265.9) | *0.18* |
| Asparagine | 2.8 (18.3) | -1.5 (19.0) | 0.60 | 0.1 (6.3) | 8.3 (10.8) | *0.31* |
| Aspartate | -23.9 (15.7) | -29.2 (15.6) | 0.45 | -23.6 (14.0) | -24.7 (5.3) | 0.82 |
| Choline | -5.2 (13.4) | -7.4 (14.3) | *0.27* | -6.5 (17.0) | 0.3 (3.4) | 0.76 |
| Cysteine | 19.6 (27.3) | 30.1 (52.9) | 0.58 | 12.9 (23.6) | 10.0 (21.1) | 0.18 |
| Cystine | 0.9 (37.5) | 5.2 (61.7) | 0.78 | -42.6 (31.9) | -2.8 (21.5) | 0.005* |
| Formate | 34.6 (21.1) | 53.5 (41.0) | *0.31* | 40.5 (18.2) | 40.3 (21.5) | 0.98 |
| Glucose (ΔmM) | -0.625 (376.8) | -1.015 (0.840) | 0.22 | -0.35 (1.2) | -0.27 (0.91) | 0.85 |
| Glutamate | 1.7 (72.6) | -2.4 (35.5) | 0.54 | 1.3 (64.0) | -10.2 (45.8) | *0.85* |
| Glutamine | -8.1 (467.9) | 34.9 (28.6) | 0.94 | -86.8 (380.4) | 105.3 (203) | 0.18 |
| Glycerol | 84.5 (37.0) | 157.3 (62.6) | 0.005* | 79.4 (33.5) | 128.7 (45.6) | 0.016* |
| Glycine | 56.2 (40.3) | 41.7 (52.5) | *0.19* | 39.6 (34.1) | 47.2 (28.5) | 0.6 |
| *Histidine* | 39.8 (16.1) | 33.8 (10.1) | 0.33 | 28.9 (9.9) | 33.9 (14.9) | 0.40 |
| Hypoxanthine | 0.3 (10.4) | 3.8 (9.6) | 0.45 | 3.5 (7.2) | -2.9 (6.5) | 0.055 |
| Inosine | 1.1 (2.8) | -0.2 (1.1) | *0.59* | N/A | | |
| *Isoleucine* | 25.3 (48.3) | -30.5 (25.2) | *0.002 ** | -9.7 (41.6) | -29.2 (71.8) | 0.48 |
| Lactate (ΔmM) | 1.4 (0.6) | 1.5 (0.5) | 0.35 | 1.8 (1.5) | 1.8 (0.9) | *0.29* |
| *Leucine* | 22.6 (54.3) | -38.0 (40.8) | 0.011* | 3.8 (55.9) | -9.8 (78.6) | 0.66 |
| *Lysine* | 29.4 (139.1) | -8.4 (64.5) | 0.97 | 17.6(159.7) | 9.7 (50.5) | *0.24* |
| *Methionine* | -0.5 (15.1) | -8.9 (8.3) | *0.16* | -3.2 (12.4) | -1.7 (10.2) | *0.54* |
| Niacinamide | 15.8 (4.1) | 12.9 (3.5) | 0.12 | 16.3 (6.9) | 11.9 (2.4) | 0.08 |
| *Phenylalanine* | 26.5 (19.6) | 21.5 (17.9) | 0.56 | 22.8 (10.6) | 19.7 (13.5) | 0.57 |
| Proline | -11.2 (44.1) | 38.8 (178.2) | *0.73* | -54.1 (56.8) | 13.9 (36.4) | 0.007* |
| Pyroglutamate | -123.3 (300.2) | -242.3 (244.0) | 0.34 | -160.9 (196.3) | -172.2 (252.3) | 0.91 |
| Pyruvate | -332.5 (128.2) | -188.9 (329.2) | *0.24* | -344.0 (107) | -226.1 (100) | 0.02* |
| Serine | -129.8 (42.0) | -102.4 (79.5) | 0.34 | -120.7 (82.5) | -90.5 (91.5) | 0.46 |
| Threonine | 119.6 (56.3) | 77.6 (58.6) | *0.19* | 106.1(82.2) | 69.1 (51.6) | 0.25 |
| Tryptophan | 0.5 (4.0) | -1.5 (2.8) | *0.63* | -4.5 (4.6) | -1.4 (1.9) | 0.07 |
| Tyrosine | 54.9 (34.3) | 39.0 (23.7) | *0.43* | 48.2 (17.5) | 52.0 (10.9) | *0.18* |
| *Valine* | 103.2 (63.9) | 52.8 (49.3) | *0.04** | 83.6 (52.8) | 66.0 (43.7) | 0.43 |

**Table S2) Conditioned media metabolite exchange**

Conditioned media metabolites as assessed by NMR in paired omental and subcutaneous adipose tissue explants from IIH and control patients, where values are relative to a non-adipose incubated blank. Statistical significance is denoted by *, where p<0.05 is considered significant. Numbers marked in *Italics*= Mann-Whitney U-test, unmarked = unpaired T-test. Data presented as mean±SD.
